# Book Review: The Social Nature of Emotion Expression: What Emotions Can Tell Us About the World

**DOI:** 10.3389/fpsyg.2021.686182

**Published:** 2021-05-14

**Authors:** Yonghe Ti, Jiangfeng Yang

**Affiliations:** ^1^Institute of Education, Tsinghua University, Beijing, China; ^2^College of Education, Zhejiang University, Hangzhou, China

**Keywords:** emotion expression, social act, expresser-observer interaction, reverse engineering, bidirectional influence

As the title suggests, this book focuses on the understanding of emotion expression in an authentic social context. Emotion expression in this book refers to a social act involving expresser, observer, and social context, which means both the social context and the emotion itself have the interpretative meaning for social agents. The explanation of emotion expression in this book not only changes some previous understanding of emotion communication in emotion science but also shows the pioneering efforts of researchers to understand emotion expression in a social context.

This book comprises 13 chapters. Chapter 1 outlines the contents of this book and introduces the emotion-based inference in context model (EBIC) as the foundation to interpret emotion expression in the social context. The rest of the chapters can be divided into four thematic groups. The first thematic group elaborates the research challenges and initiations of emotion recognition (Chapter 2) and emotion perception (Chapter 3) in real social interactions. The second thematic group explains the bidirectional influence between social context and emotional expression, which is the core part of this book. Specifically, three processes, reverse engineering (Chapter 6), reverse appraisal (Chapter 8), and social referencing (Chapter 7 and 11), are described to illustrate the reconstruction of appraisal of emotion in social context. The third thematic strand explains the interpretable meaning of emotion expression in the social context which is the communicative signals (Chapter 4 and 5) and inferential cues of the expresser's personality and social status (Chapter 9 and 10). The fourth thematic group focuses on the social meaning of specific facial emotional expressions such as cry (Chapter 12) and smile (Chapter 13).

The reason why this book is attractive is that emotional expression is understood in a dynamic and interactive social context in each chapter, changing the ideas that focus on the basic elements of emotional expression in most previous studies.

This book especially focuses on the instrumental role of emotional expression in transmitting information on social interaction. According to the previous definition, emotional expression is pre-programmed, unintended, and involuntary, even if life experiences shape it (Ekman and Cordaro, 2011). Nevertheless, in this book emotional expression is described as “a means of expressing inner states, of representing what the world is like, of directing other people's behavior, and of committing to future courses of action” (p. 49). It shows emotional expression is influenced by social motives and not even necessarily authentic. As stated in chapter 13, although it is believed that smile is an external expression of enjoyment or satisfaction, people sometimes smile to mask their negative feelings in front of others. That is to say, rather than in the static or completely steerable environments, understanding diversity and inhomogeneity of emotion expression in authentic environments may be more valuable to enhance comprehension of our daily social intentions and behaviors.

This book emphasizes the perspective of observers toward emotion expression in complex social interaction. In the light of the EBIC model of this book, observers receive the information underlying emotion expression and match it to social context with their motivations and goals in order to react while perceiving expresser's emotion, which means emotion expressions impact both the expressers (intrapersonal effect) and the observers (interpersonal effect). Appraisal theory of emotions and its reverse deduction compose a loop conjointly to explicate the different agents' tendencies in social interaction. Although the reverse explanation of emotion expression has been advancing, this book also points out that what inferences are the outcome of reverse engineering and whether the reverse inferences are automatic of observers remain an open question for future research (p. 114).

In addition, several chapters of this book critically inspect the current methods to study emotion expression in the social context. For example, Chapter 2 proposes that traditional research on emotion recognition mostly depends on the match-mismatch paradigm, failing to recognize the social meaning of emotion. Besides, some measurements may not capture effectively the relevancy between emotion ability and social competencies because of the lack of sensitivity to social situations. Therefore, with the trend of interdisciplinary and applied research on emotion science (Calvo and D'Mello, 2010; Juckel et al., 2018), new tools need to be explored and applied for emotion expression research in the authentic and tangible social context.

Overall, this is a legible yet insightful book that should not be missed. Potential readership refers to the theoretical or applied researchers of emotion science, for this book provides a model to understand emotion expression in the social context, which may even conduce to develop emotional intelligent robots in the future (p. 154). Moreover, reading this book can also be beneficial from a naïve perspective. It can serve as a mirror for ordinary readers to scrutinize the emotion expression in daily life, so that they may realize the information hidden behind the seemingly meaningless emotion expression in social interaction.

## Author Contributions

YT has written the review. JY revised the drafts. Both authors contributed to the article and approved the submission for publication.

## Conflict of Interest

The authors declare that the research was conducted in the absence of any commercial or financial relationships that could be construed as a potential conflict of interest.
